# A Convolutional Neural Network with Spatial Location Integration for Nearshore Water Depth Inversion

**DOI:** 10.3390/s23208493

**Published:** 2023-10-16

**Authors:** Chunlong He, Qigang Jiang, Guofang Tao, Zhenchao Zhang

**Affiliations:** College of Geoexploration Science and Technology, Jilin University, Changchun 130026, China; hecl22@mails.jlu.edu.cn (C.H.); taogf18@mails.jlu.edu.cn (G.T.); zhangzc19@mails.jlu.edu.cn (Z.Z.)

**Keywords:** convolutional neural network, spatial location information, water depth inversion, remote sensing

## Abstract

Nearshore water depth plays a crucial role in scientific research, navigation management, coastal zone protection, and coastal disaster mitigation. This study aims to address the challenge of insufficient feature extraction from remote sensing data in nearshore water depth inversion. To achieve this, a convolutional neural network with spatial location integration (CNN-SLI) is proposed. The CNN-SLI is designed to extract deep features from remote sensing data by considering the spatial dimension. In this approach, the spatial location information of pixels is utilized as two additional channels, which are concatenated with the input feature image. The resulting concatenated image data are then used as the input for the convolutional neural network. Using GF-6 remote sensing images and measured water depth data from electronic nautical charts, a nearshore water depth inversion experiment was conducted in the waters near Nanshan Port. The results of the proposed method were compared with those of the Lyzenga, MLP, and CNN models. The CNN-SLI model demonstrated outstanding performance in water depth inversion, with impressive metrics: an RMSE of 1.34 m, MAE of 0.94 m, and R^2^ of 0.97. It outperformed all other models in terms of overall inversion accuracy and regression fit. Regardless of the water depth intervals, CNN-SLI consistently achieved the lowest RMSE and MAE values, indicating excellent performance in both shallow and deep waters. Comparative analysis with Kriging confirmed that the CNN-SLI model best matched the interpolated water depth, further establishing its superiority over the Lyzenga, MLP, and CNN models. Notably, in this study area, the CNN-SLI model exhibited significant performance advantages when trained with at least 250 samples, resulting in optimal inversion results. Accuracy evaluation on an independent dataset shows that the CNN-SLI model has better generalization ability than the Lyzenga, MLP, and CNN models under different conditions. These results demonstrate the superiority of CNN-SLI for nearshore water depth inversion and highlight the importance of integrating spatial location information into convolutional neural networks for improved performance.

## 1. Introduction

Nearshore water depth is a crucial geophysical parameter in the coastal environment, with significant importance for scientific research, navigation management, coastal zone protection, and coastal disaster mitigation [1,2,3]. Traditional methods for acquiring water depth data have primarily involved single-beam and multibeam sonar soundings, as well as airborne LiDAR measurements. While these methods provide high accuracy in obtaining water depth information, they are limited by the prevailing climatic conditions at the measurement sites, often require substantial human and material resources, and are insufficient for capturing extensive water depth information. Moreover, regions that are inaccessible to ships or airborne platforms, such as dangerous waters with reefs or islands involved in political disputes, present challenges for using traditional methods to collect water depth data [4,5,6,7]. In recent years, there has been significant attention given to research on water depth inversion using satellite remote sensing. The use of satellite remote sensing for water depth inversion offers several advantages in terms of extensive coverage, cost-effectiveness, rapid data acquisition, and ease of remote sensing image acquisition. Additionally, this approach allows for observations of areas that are inaccessible to ships or airborne platforms, thus providing promising prospects for development and application [8,9]. Previous studies in this field have led to numerous achievements in estimating shallow water depth using satellite remote sensing images. These efforts can be broadly categorized into semi-analytical models, empirical models, and machine learning models [10,11,12].

A semi-analytical model is developed based on the physical radiative transfer theory of visible light in water bodies. It establishes analytical expressions that connect spectral reflectance with bottom reflectance, water depth, and inherent optical properties (IOPs). The primary objective of this model is to minimize the discrepancy between observed and simulated spectral reflectance, thus enabling the inversion of water depth and IOPs. For example, Lee et al. utilized the Hyperspectral Optimization Process Exemplar (HOPE) algorithm and Hyperion hyperspectral data to estimate water depth and IOPs in the Florida Keys region. They observed a relative error of 11% in the inverted water depth compared to LiDAR measurements [13]. Similarly, Wei et al. employed marine color satellite data and temporal variations in water column IOPs from two satellite measurements to perform water depth inversion. Their results demonstrated the accuracy of this method in estimating depth within the range of 0–30 m [14]. Another approach was introduced by Xia et al., who developed the L-S model by integrating the log-ratio model with the semi-analytical model. They used remote sensing data from four bands to estimate the water depth around Ganquan Island in the South China Sea. Interestingly, the study found that the L-S model yielded comparable results to those of the log-ratio model, even without utilizing actual depth measurements [15]. Zhang et al. improved the HOPE algorithm by utilizing Hyperion hyperspectral images to estimate water depth around Saipan Island and Zhongye Island. This enhancement led to significantly improved inversion results, addressing the issue of overestimating water depth in low-reflectance areas encountered by traditional HOPE algorithms [16]. From the above research, the semi-analytical model demonstrated robust physical interpretability and eliminated the need for empirical depth data. However, it involved more than five unknown parameters related to bottom reflectance and IOPs. Hyperspectral images, with their high spectral resolution across the visible light wavelength range, were found to be more suitable than multispectral images for resolving these unknown parameters in the modeling context. Nonetheless, acquiring appropriate hyperspectral images of target regions remained a challenge, often limited by spatial resolution.

Empirical models require in situ water depth measurements to establish a statistical relationship between spectral reflectance and actual water depth. For example, Lyzenga employed empirical regression analysis to develop expressions that relate image reflectance to measured water depth, enabling remote sensing-based water depth inversion. The study demonstrated the model’s remarkable applicability in shallow water depth inversion [17]. Paredes proposed a two-band log-linear model based on a constant ratio of reflectance between two spectral bands on different seafloor substrates. This model was later extended to multiple bands and has been widely recognized for its superiority over single-band counterparts [18]. Stumpf further enhanced the log-transformed ratio model, which showed improved stability and higher inversion accuracy in turbid waters compared to linear models [19]. These empirical models were found to be relatively simple to establish and required only a modest collection of water depth points to extrapolate information over large marine areas. However, it should be noted that the performance of these models is highly influenced by the complexity of the aquatic environment within the study area.

In recent years, several studies have focused on using shallow machine learning techniques to invert water depth in specific marine regions. For instance, Zheng et al. utilized WorldView-2 remote sensing images as their data source and developed two artificial neural network models, namely Back Propagation (BP) and Radial Basis Function (RBF), for water depth inversion at Meiji Reef in the South China Sea. The results indicated that the RBF neural network model was more suitable for water depth inversion [20]. Sagawa et al. employed multi-temporal Landsat-8 remote sensing images and a random forest model to accurately invert water depth in highly transparent coastal waters [21]. Similarly, Sun et al. used Gaofen-6 remote sensing images and applied a support vector regression algorithm along with two regression tree models to invert shallow water depth in the southern part of the Shandong Peninsula. The experimental results demonstrated that the two regression tree models exhibited superior inversion accuracy [22]. From the above research, it is worth noting that the effectiveness of shallow water depth inversion has been significantly limited by the reliance on manual expertise or feature transformation techniques to extract remote sensing features.

Currently, most semi-analytical, empirical, and shallow machine learning models usually obtain water depth information based on the relationship between the spectral reflectance of individual pixels and the measured water depth. However, the limited amount of information provided by individual pixels in spectral reflectance images is constantly affected by sensor noise, imprecise atmospheric correction, and undulating water surfaces. Despite these limitations, there is still significant potential for improving the effectiveness of nearshore water depth inversion [23,24]. Some studies have utilized multi-temporal satellite data to create time series, increasing the dimensions of the data and enhancing the fitting of the nonlinear relationships between satellite data and water depth [21,25,26]. However, the constraints imposed by revisit cycles and potential cloud cover can hinder the acquisition of temporally close and high-quality remote sensing data in the target region, thus limiting the feasibility of this approach for water depth inversion. The extraction of feature information from satellite remote sensing data relevant to water depth signals while considering the effects of complicated nonlinear factors remains a significant challenge in nearshore water depth inversion research.

Deep learning methods, particularly convolutional neural networks (CNNs), have been extensively utilized in remote sensing image processing [27,28]. CNNs have shown great proficiency in feature extraction and integration, making them highly effective in various domains such as image classification, object detection, and change detection [29]. In addition to remote sensing image processing, CNNs have also been explored for remote sensing surface parameter inversion [30,31]. By utilizing stacked convolutional and pooling layers, CNNs automatically capture crucial spectral and spatial features from remote sensing data [32]. Their ability to integrate multiple features through fully connected layers makes them well suited for nearshore water depth inversion research. The inclusion of spatial location information of pixels plays a significant role in enhancing the accuracy of empirical methods and shallow machine learning techniques for water depth inversion [33,34,35,36,37]. However, there is a lack of conclusive evidence regarding the impact of spatial location information on the results of water depth inversion using CNNs. In many cases, the spatial location information of pixels is disregarded when applying CNNs to water depth inversion [38,39,40]. Despite CNNs inherently possessing the capability to capture local spatial correlations among neighboring pixels, the inclusion of spatial location information could potentially enhance the performance of water depth inversion using CNNs.

To address the challenge of extracting feature information from remote sensing data for nearshore water depth inversion, this study proposes a novel approach called the convolutional neural network with spatial location integration (CNN-SLI). The CNN-SLI integrates spatial location information by adding two additional channels that represent the pixel locations to the input feature image. These concatenated image data are then fed into the CNN, allowing it to better capture and understand spatial variations. By doing so, the proposed method aims to mitigate the loss of spatial information caused by successive convolution and pooling operations, ultimately improving the accuracy of water depth inversion. The water depth inversion experiment was conducted in the waters near Nanshan Port. The experiment utilized a GF-6 remote sensing image and measured water depth data from electronic nautical charts. To evaluate the effectiveness of the CNN-SLI model, a comparative analysis was performed between the water depth inversion results obtained using the proposed model and those obtained using the Lyzenga, MLP, and regular CNN models.

The innovation of this article is to consider additional feature information, that is, the spatial location information of pixels when using a CNN to invert water depth. At present, there are few studies that prove that the spatial location information of pixels is helpful for water depth inversion of CNNs because CNNs already have the ability to capture the local spatial correlation of adjacent pixels. This study confirms that considering the spatial location information of pixels can effectively improve the water depth inversion performance of CNNs.

## 2. Study Area and Data

### 2.1. Study Area

**Figure 1:**
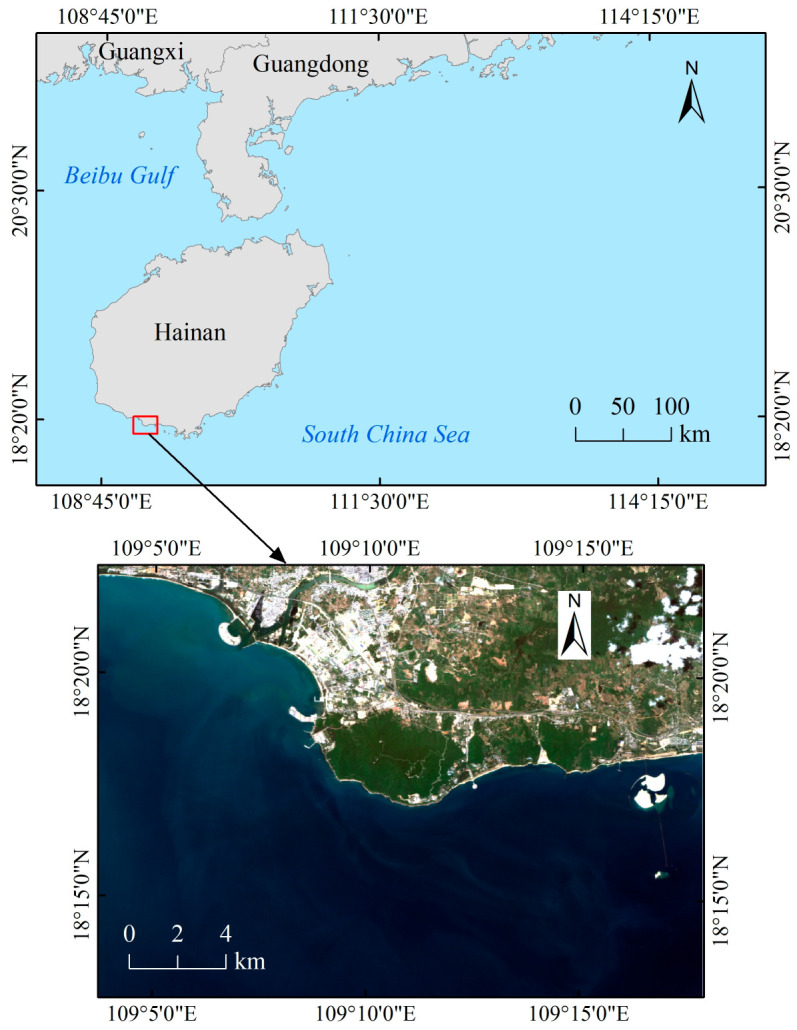
Geographic location of the study area.

The study area is located in the southwest of Hainan Island, China, specifically in the waters near Nanshan Port (Figure 1). It is in close proximity to human settlements and experiences a high volume of maritime traffic. The water quality of this water area is poor, and the substrate type is single, so it is suitable as an experimental area for remote sensing water depth inversion.

### 2.2. Data

The multispectral remote sensing data used in this paper is the Gaofen-6 wide-field-of-view (WFV) remote sensing image from the China Centre for Resources Satellite Data and Application (https://data.cresda.cn, accessed on 22 March 2023). The Gaofen-6 satellite was launched on 2 June 2018. It is China’s first optical remote sensing satellite carrying red-edge band information. It is equipped with a high-resolution PMS sensor and a wide-field WFV sensor, providing high spatial resolution, extensive coverage, and short revisit intervals. Gaofen-6 WFV remote sensing data have eight spectral bands with 16 m spatial resolution. For brevity, the Gaofen-6 WFV remote sensing image will be referred to as GF-6. The key parameters of GF-6 can be found in Table 1.

The measured water depth data come from two electronic navigation charts (ENC) in S-57 format released by the Maritime Safety Administration of the People’s Republic of China (https://www.chart.msa.gov.cn, accessed on 5 June 2023), with chart numbers CN403301 and CN303002, respectively. The ENC with drawing number CN403301 is abbreviated as ENC-A, and the ENC with drawing number CN303002 is abbreviated as ENC-B. The water depth data in ENC-A are recorded as Dataset A and were mainly measured in April 2021. The water depth data in ENC-B, denoted as Dataset B, were mainly measured before 2010 and were used as an independent dataset to test the generalization ability of different models. Dataset A/B complies with the Category of Zone of Confidence (CATZOC) standard proposed by the International Hydrographic Organization (IHO) and meets the ZOC-B standard. The bathymetry error of Dataset A/B is 1.2 m in the water depth range of 0–10 m and 1.6 m in the water depth range of 10–30 m.

## 3. Research Methods

**Figure 2:**
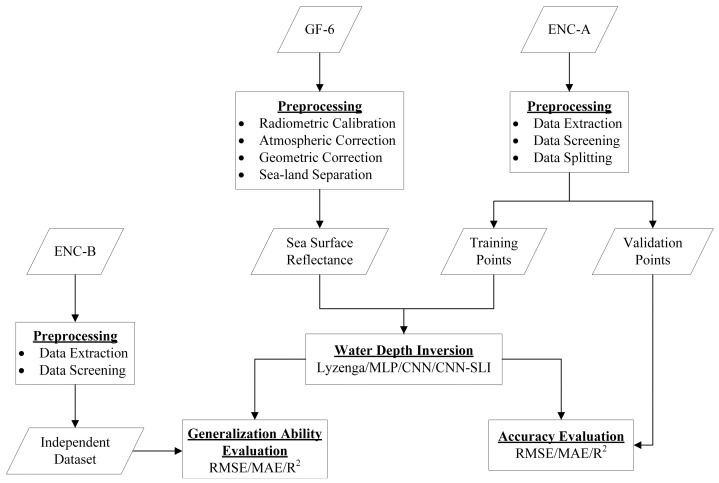
Workflow chart of water depth inversion.

This section details the processing steps involved in the water depth inversion process and the applied inversion methods. Figure 2 shows the workflow of water depth inversion.

### 3.1. Data Preprocessing

#### 3.1.1. Preprocessing of Measured Water Depth Data

**Figure 3:**
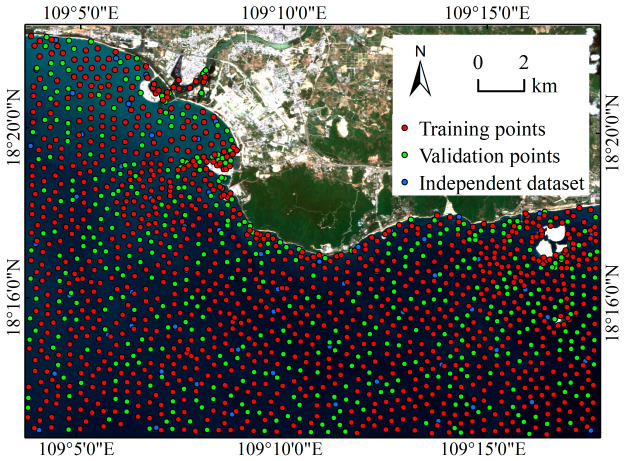
Distribution of measured water depth points in the study area.

Global Mapper version 20 software was used to extract 1512 water depth points from ENC-A, which were evenly distributed in the study area. However, 45 water depth points are not distributed in the waters of the study area, and one water depth point has numerical anomalies. Therefore, Dataset A has a total of 1466 water depth points. A total of 67 water depth points located within the study area were extracted from ENC-B, so Dataset B has a total of 67 water depth points. After that, the training samples and test samples were split from Dataset A according to the ratio of 7:3 through the subset features tool of the ArcGIS software. Dataset B is used as an independent dataset to test the generalization ability of different models. The spatial distribution of water depth points is shown in Figure 3. The statistical information of water depth data is shown in Table 2.

#### 3.1.2. Data Preprocessing of Remote Sensing Data

Prior to constructing water depth inversion models, several preprocessing steps are necessary for remote sensing images. These steps include radiometric calibration, atmospheric correction, and geometric correction. Radiometric calibration involves converting grayscale values into physical quantities like apparent radiance or reflectance, which helps correct errors in the sensor output. In this study, the radiometric calibration tool embedded in the ENVI software was used to convert the grayscale values of the GF-6 remote sensing image to upper atmospheric apparent radiance. Atmospheric correction aims to mitigate the effects of atmospheric scattering and aerosols. For this purpose, the FLAASH atmospheric correction algorithm, based on the MODTRAN 4+ radiative transfer model, was employed to convert the upper atmospheric apparent radiance of the GF-6 remote sensing image to the lower atmospheric surface reflectance. The FLAASH algorithm, now integrated into the ENVI software, offers a user-friendly interface that facilitates straightforward visual operations. Following atmospheric correction, remote sensing images usually undergo geometric correction to address geometric distortions caused by the image acquisition process. The ENVI software’s RPC orthorectification tool was used to correct the GF-6 image.

Afterward, following the principles of typical, full-scale, and even distribution, samples (separability greater than 1.9) were selected through visual interpretation, and the selected samples and the support vector machine algorithm embedded in the ENVI software were used to mark land and ocean areas. A land mask is then generated. The function of the land mask is to mask out the land part in the water depth inversion results.

### 3.2. Water Depth Inversion Methods

#### 3.2.1. Traditional Water Depth Inversion Methods

- Log-Linear Model

The log-linear model (Lyzenga) is a widely used empirical model with mathematical expression [17]. It is a straightforward and effective model, as demonstrated in Equation (1).

(1) $$Z=a_{0}+\sum_{i=1}^{N}\left\{a_{i}*\ln\left[R\left(\lambda_{i}\right)-R_{\infty }\left(\lambda_{i}\right)\right]\right\},$$

Here, Z is the water depth, $a_{0}$ and $a_{i}$ are the regression coefficients of the model, N is the number of bands involved in the inversion, $R\left(\lambda_{i}\right)$ is the reflectance of band i, and $R_{\infty }\left(\lambda_{i}\right)$ is the reflectance of band i in deep water. In this paper, the Lyzenga model uses the reflectance values of all eight bands of GF-6.

- Multilayer Perceptron

**Figure 4:**
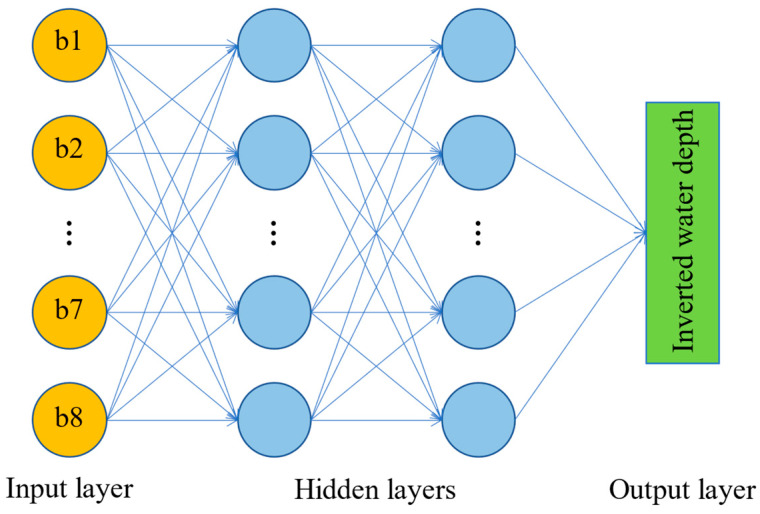
Schematic structure of the MLP model.

The multilayer perceptron (MLP) is a feedforward neural network commonly used in machine learning. It consists of an input layer, an output layer, and multiple hidden layers [41]. The adjacent layers are connected through weights and activation functions. The number of neurons in the input layer corresponds to the number of input features. In this article, the reflectance values of all eight bands of GF-6 are used as the input data of the MLP model, so the number of neurons in the input layer is 8. The output layer provided the water depth inversion results and had a single neuron. The MLP regression algorithm with two hidden layers was implemented using the PyTorch version 1.10.1 framework in the Python programming language. Figure 4 illustrates the structure of the MLP model. The ReLU function was chosen as the activation function for the hidden layers, and the Adam algorithm was selected as the weight optimization solver. To mitigate the risk of overfitting, a dropout layer (enabled only during training) is added behind each hidden layer, and the dropout probability is empirically set to 0.1. To determine the batch size, learning rate, number of iterations, and number of neurons in the hidden layers, Bayesian search and 5-fold cross-validation methods were employed with the Scikit-Optimize library. The prescribed values for the hyperparameters within the MLP model are shown in Table 3.

#### 3.2.2. CNN Model

The Convolutional Neural Network (CNN) is a deep feedforward neural network that incorporates local connectivity and weight sharing. It effectively utilizes both spectral and spatial information present in remote sensing images, making it applicable in various domains such as remote sensing image classification, object detection, and change detection [29]. In addition to remote sensing image processing, CNNs have also been explored for remote sensing surface parameter inversion [30,31]. A typical CNN consists of convolutional layers, pooling layers, and fully connected layers, with the fully connected layers following the convolutional and pooling layers [40]. Convolutional layers use kernels to extract local features from the input data while pooling layers reduce the dimensionality of the features to prevent overfitting. Fully connected layers refine and integrate the extracted features, and the final layer produces predictions. Activation functions introduce nonlinear mapping capabilities to both convolutional and fully connected layers. In general, for CNNs, the unnormalized outputs are typically passed through the softmax function to obtain class probabilities [41]. However, in the context of this paper, where the CNN output predicts water depth, it is appropriate to bypass the softmax function.

**Figure 5:**
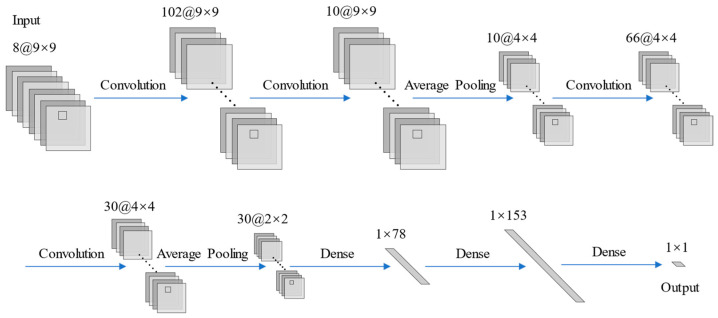
Structure of the CNN model.

The CNN algorithm in this study was implemented using the Pytorch framework in the Python programming language. The structure of the constructed CNN is illustrated in Figure 5, consisting of four convolutional layers, two pooling layers, and three fully connected layers. The last fully connected layer served as the output layer, responsible for outputting the inverted water depth value with a single neuron. The input layer of the CNN required a normalized patch image (9 × 9), which was obtained by cropping the preprocessed remote sensing image using the spatial coordinates of the water depth points and then processed using the Zero-Score normalization method. Since GF-6 remote sensing images have limited spatial resolution, a 3 × 3 convolution kernel size was chosen to effectively utilize the spatial information of neighboring pixels. To prevent information loss at the edges of the image during the convolution operation, a padding operation was performed in the convolution layer. The pooling operation was performed using an average pooling layer with a kernel size of 2 × 2 and a stride size of 2. The optimizer chosen was the Adam function, with ReLU as the activation function. The mean square error (MSE) was used as the loss function for the output layer. To mitigate the risk of overfitting, except for the output layer, a dropout layer (enabled only during training) is added behind other fully connected layers, and the dropout probability is empirically set to 0.1. Bayesian search and 5-fold cross-validation methods were implemented using the Scikit-Optimize library to determine the batch size, learning rate, number of iterations, number of convolution kernels, and number of neurons in the fully connected layers. The prescribed values for the hyperparameters within the CNN model are shown in Table 4.

#### 3.2.3. Implementation of CNN-SLI Model

Generally, a CNN takes as an input the spectral reflectance of neighboring pixels centered on the water depth point, which is a patch image cropped from the spectral reflectance image [26,38,40]. Existing CNN models rely on data mining to establish a nonlinear mapping between water depth and spectral reflectance images while disregarding the impact of spatial location information. Previous studies have demonstrated that incorporating spatial location information of pixels can significantly improve the accuracy of empirical methods and shallow machine learning methods for water depth inversion [33,34,35,36,37]. However, it has not yet been proven whether the spatial location information of pixels is also beneficial for the water depth inversion of CNN models. Although CNN models already have the capability to capture the local spatial correlation of neighboring pixels, the inclusion of spatial location information may still contribute to the water depth inversion accuracy of CNN models.

In this study, we propose a convolutional neural network with spatial location integration (CNN-SLI) to improve the accuracy of nearshore water depth inversion using remote sensing data. The CNN-SLI model integrates the normalized spatial location information (X and Y coordinates) as additional channels, which are concatenated with the normalized patch image (9 × 9). These concatenated image data are then used as the input to the CNN-SLI model, allowing it to better capture spatial information variations and mitigate the loss of spatial information caused by successive convolutional and pooling operations.

**Figure 6:**
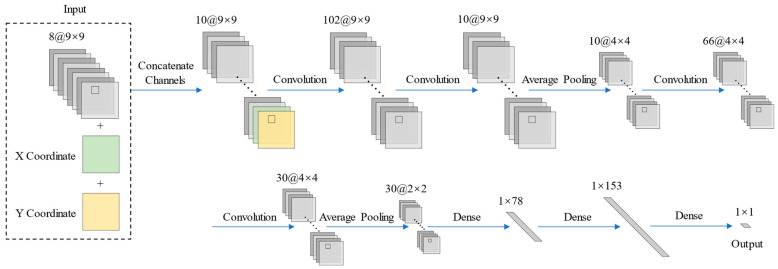
Structure of the CNN-SLI model.

The network structure and parameter settings of the CNN-SLI model are similar to those of the CNN model, with the only difference being the input layer of the CNN-SLI model, which requires the input of the concatenated image data generated from the normalized spatial location information and the normalized patch image in the channel dimension. Figure 6 illustrates the structure of the CNN-SLI model.

### 3.3. Accuracy Evaluation Indicators

The root mean square error (RMSE), the mean absolute error (MAE), and the coefficient of determination (R^2^) are used to evaluate the accuracy of the water depth inversion results. The RMSE and MAE are used to evaluate the error between the inverted water depth and the measured water depth, and the smaller the values, the more accurate the water depth inversion results are. R^2^ reflects the degree of fit between the inverted water depth and the measured water depth, with a value in the range [0, 1], and the closer the value is to 1, the better the water depth inversion results are.

(2) $$\mathrm{RMSE}=\sqrt{\frac{\sum_{i=1}^{n}{\left(y_{i}-y_{i}^{T}\right)}^{2}}{n}},$$

(3) $$\mathrm{MAE}=\frac{\sum_{i=1}^{n}\left|y_{i}-y_{i}^{T}\right|}{n},$$

(4) $$R^{2}=\frac{\sum_{i=1}^{n}{\left(y_{i}-\bar{y}\right)}^{2}}{\sum_{i=1}^{n}{\left(y_{i}^{T}-\bar{y}\right)}^{2}},$$

Here, $y_{i}$ and $y_{i}^{T}$ are the inverted water depth and measured water depth of the ith validation sample, respectively, $\bar{y}$ is the average of the measured water depth of the validation samples, and n is the total number of validation samples.

## 4. Results and Discussion

### 4.1. Maps of Inverted Water Depth

**Figure 7:**
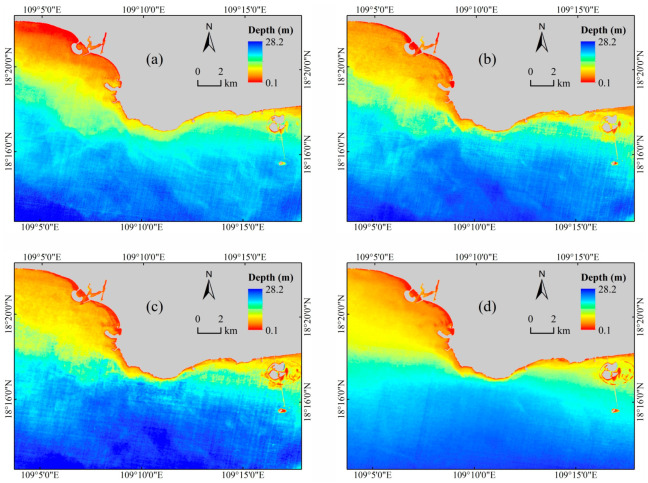
Inverted water depth maps for Nanshan Port. (**a**) Lyzenga model. (**b**) MLP model. (**c**) CNN model. (**d**) CNN-SLI model.

The inverted water depth maps of the waters near Nanshan Port were generated using trained models and the preprocessed GF-6 remote sensing image, and Figure 7 shows the results. The values on the maps represent the water depth, with larger values corresponding to deeper waters. Figure 7a–d shows the inverted water depth maps obtained by the Lyzenga, MLP, CNN, and CNN-SLI models, respectively. The gray area is the generated land mask. As depicted in Figure 7, the spatial distributions of inverted water depth obtained by the four inversion models are roughly similar. All the inverted water depth maps exhibit a gradual increase in water depth from the coastline to the open sea. Due to the uneven distribution of water quality elements such as chlorophyll and suspended sediment caused by the fluctuation of the water surface, there are some cyan areas with a fuzzy appearance similar to mist in the inverted water depth maps of the Lyzenga, MLP, and CNN models, while there is no such phenomenon in the inverted water depth map of the CNN-SLI model. There are underwater buildings in the area on the right side of the figure. It can be seen that the water depth maps inverted by the CNN and CNN-SLI models more accurately reflect the underwater topography of this area than the Lyzenga and MLP models. It can also be found that the inverted water depth maps of the CNN and CNN-SLI models have less speckle noise than the other two models, whether in the blue deep waters or the red shallow waters.

### 4.2. Overall Accuracy Evaluation

This paper evaluates the inversion effectiveness of four models (Lyzenga, MLP, CNN, and CNN-SLI) by calculating three accuracy evaluation indicators (RMSE, MAE, and R^2^) using the validation dataset. Table 5 presents a comparison of the overall inversion accuracy achieved by different water depth inversion methods. The CNN-SLI model demonstrates the highest overall accuracy, with an RMSE of 1.34 m and an MAE of 0.94 m. This represents a significant improvement compared to the CNN model, with a reduction of 0.59 m in the RMSE and 0.52 m in the MAE. The CNN model follows with the second-best overall accuracy, achieving an RMSE of 1.93 m and an MAE of 1.46 m. The overall inversion accuracy of the MLP model is slightly lower than the CNN model but significantly better than the Lyzenga model, with an RMSE of 2.04 m and an MAE of 1.55 m. The Lyzenga model exhibits the poorest overall inversion performance, with an RMSE of 2.68 m and an MAE of 2.08 m.

It can also be seen from Table 5 that for processing a GF-6 image (size: 1561 × 1118), the comparative results of the time required by each model are as follows: Lyzenga (2.44 s) < MLP (66.89 s) < CNN (112.12 s) < CNN-SLI (237.84 s). The CNN-SLI model has the longest running time, but this is not an obstacle to the application and promotion of the algorithm because, in some real scenarios, the importance of inversion accuracy may be greater than the running efficiency of the algorithm.

**Figure 8:**
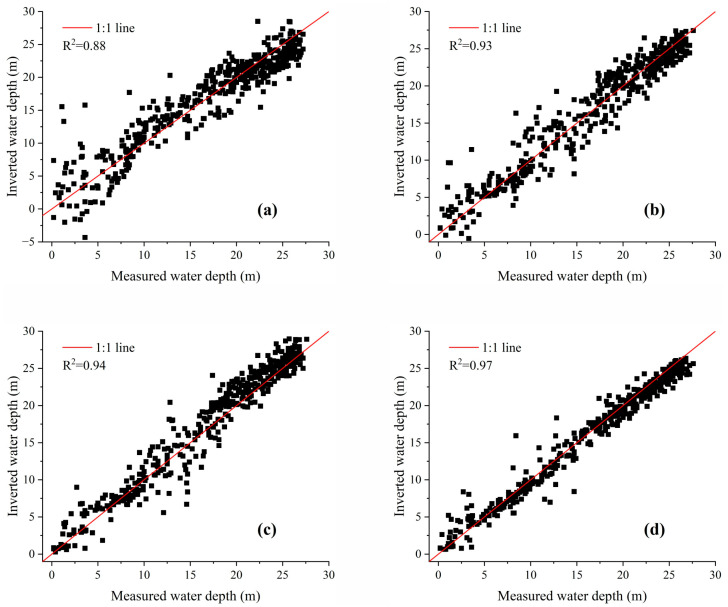
Scatter plots of inverted water depth and measured water depth for Nanshan Port. (**a**) Lyzenga model. (**b**) MLP model. (**c**) CNN model. (**d**) CNN-SLI model.

To visually assess the inversion performance, Figure 8 displays scatter plots of the four water depth inversion models using the validation dataset. The horizontal axis represents the measured water depth, while the vertical axis represents the inverted water depth. A higher R^2^ value indicates better clustering of points along the 1:1 line, indicating a better regression fit for the models. Conversely, a lower R^2^ value indicates a poorer regression fit. Among the models, CNN-SLI shows the best regression fit with an R^2^ of 0.97, which is a 3.2% improvement over the R^2^ of the CNN model. The CNN model has an R^2^ of 0.94. The R^2^ of the MLP model is 0.93, which is slightly lower than the CNN model but significantly higher than the Lyzenga model (the R^2^ of the Lyzenga model is 0.88).

Figure 8 also illustrates the scatter distributions, providing further insights. It is obvious from the scatter distribution that the CNN-SLI model exhibits a higher degree of aggregation than the CNN, MLP, and Lyzenga models in different water depth ranges. Specifically, in the water depth range of 0–14 m, the CNN-SLI model shows the highest degree of aggregation, with most checkpoints distributed on both sides of the 1:1 line. In contrast, the CNN model has a significantly lower aggregation degree, while the MLP model has a lower aggregation degree than the CNN model but significantly higher than the Lyzenga model. In the water depth range of 14–21 m, the CNN-SLI model still maintains the highest aggregation degree, while the aggregation degrees of the Lyzenga, MLP, and CNN models are relatively close. In the water depth range of 21–29 m, the CNN-SLI model also shows the highest degree of aggregation, which is significantly better than the other three models, while the degree of aggregation of the CNN model is slightly lower than the MLP model but significantly higher than the Lyzenga model.

### 4.3. Accuracy Evaluation at Different Water Depth Intervals

**Figure 9:**
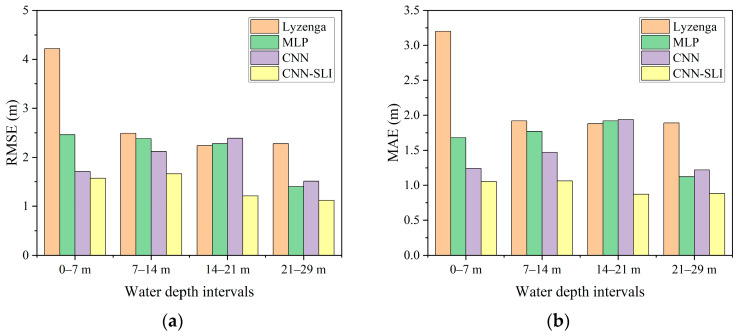
Inversion errors of different models at different water depth intervals. (**a**) RMSE. (**b**) MAE.

Figure 8 shows that different models have different inversion errors at different water depth intervals. To further evaluate the effectiveness of water depth inversion models at different intervals, the measured water depth range of the study area is divided into four intervals: 0–7 m, 7–14 m, 14–21 m, and 21–29 m. This division allows the calculation of the RMSE and MAE for each model within these intervals. The comparison of the performance of the different water depth inversion models across the intervals is presented in Table 6 and Figure 9.

As can be seen from Table 6 and Figure 9, the CNN-SLI model has the most ideal inversion results at all water depth intervals and achieves the lowest RMSE and MAE in both shallow and deep waters. The RMSE and MAE of the CNN-SLI model are 0.14–2.65 m and 0.19–2.15 m lower than other models, respectively. The performance advantage of the CNN-SLI model is particularly obvious at the water depth intervals of 7–14 m and 14–21 m. At the water depth intervals of 0–7 m and 7–14 m, the RMSE and MAE of the CNN model are lower than those of the MLP model (the RMSE is maximally lower by 0.75 m and MAE is maximally lower by 0.44 m), while at the water depth intervals of 14–21 m and 21–29 m, the RMSE and MAE of the CNN model are slightly higher than those of the MLP model (the maximum RMSE is 0.11 m higher and the MAE is maximum 0.1 m higher). The Lyzenga model achieved the highest RMSE and MAE at almost all water depth intervals and only had a slight advantage over the MLP and CNN models at the water depth interval of 14–21 m. In conclusion, the CNN-SLI model proves to be more suitable for water depth inversion in both deep and shallow waters compared to the other models.

### 4.4. Comparative Analysis between Inverted and Interpolated Water Depth

**Figure 10:**
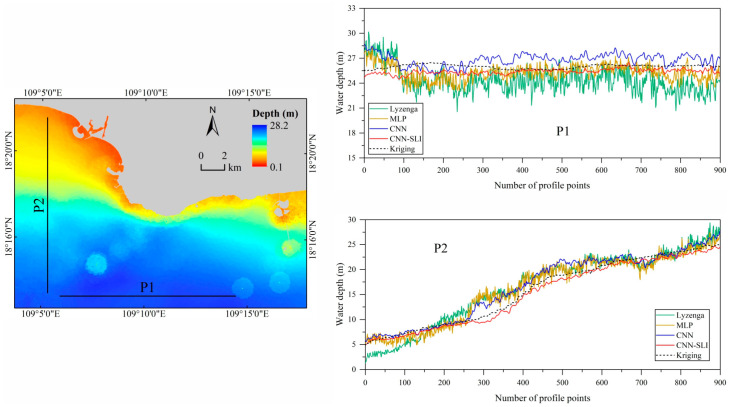
Kriging interpolation results and water depth profile results.

To better assess the inversion effect of each model, a horizontal profile and a vertical profile are selected to analyze the water depth conditions at the profiles, and the results of the water depth profiles are shown in Figure 10. The black dotted line is the Kriging interpolation results of the measured water depth points, which can be considered to be close to the actual water depth. The green solid line, brown solid line, blue solid line, and red solid line are the water depth profile results of Lyzenga, MLP, CNN, and CNN-SLI models, respectively.

As can be seen from Figure 10, the water depth profile results of the four inversion models have roughly the same trend as the Kriging interpolation results. Among them, the profile line trend of the CNN-SLI model is closest to the Kriging interpolation results. In the P1 profile, the profile line trends of the Lyzenga, MLP, and CNN models at the first 100 points show large differences compared with the Kriging interpolation results. Specifically, for the water depth estimates of the first 100 points, all three models show a tendency to overestimate. However, the trend of the CNN-SLI model in the same profile interval is closer to the Kriging interpolation result. Furthermore, in the P1 profile, the CNN model begins to overestimate the water depth value after the 250th point, while the Lyzenga model begins to underestimate the water depth value after the same position. On the contrary, the profile trend of the MLP and CNN-SLI models after the 250th point is closer to the Kriging interpolation result. It is worth noting that throughout the P1 profile, the profile lines of the Lyzenga and MLP models exhibit larger fluctuation amplitudes than the CNN and CNN-SLI models. In the P2 profile, the Lyzenga, MLP, and CNN models all overestimated the water depth value in the profile interval from the 250th point to the 600th point. The Lyzenga model also showed a trend of underestimating the water depth in the first 100 points. Only the CNN-SLI model had a trend in line with the kriging interpolation results throughout the P2 profile. Notably, the CNN and CNN-SLI models exhibit similar fluctuation amplitudes in the P2 profile, which are smaller than those of the Lyzenga and MLP models. This analysis of water depth profiles highlights the exceptional performance of the CNN-SLI model in nearshore waters, as it closely aligns with the Kriging interpolation compared to the Lyzenga, MLP, and CNN models.

**Figure 11:**
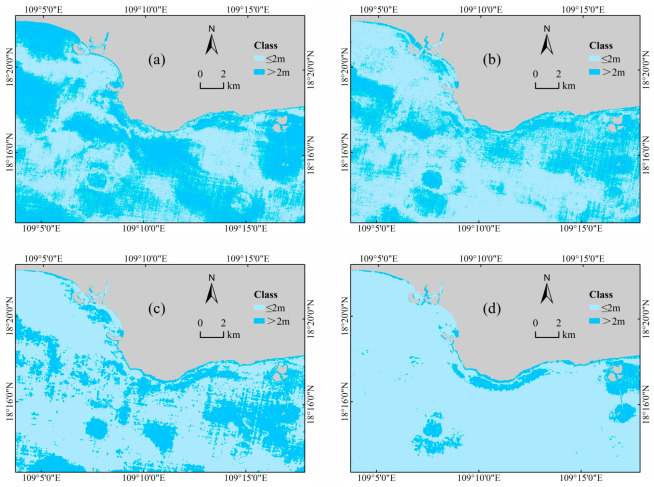
Plots of the discrepancy between inverted and interpolated water depth for different models. (**a**) Lyzenga model. (**b**) MLP model. (**c**) CNN model. (**d**) CNN-SLI model.

**Figure 12:**
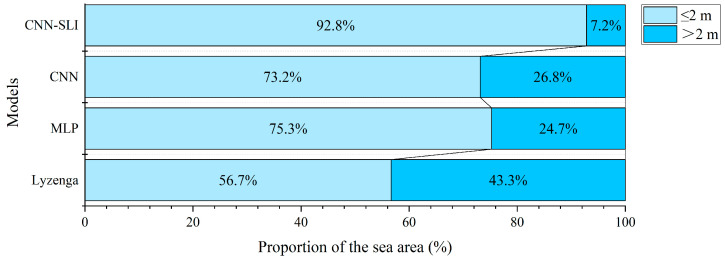
Proportion of the discrepancy range of different models to the sea area range of the entire study area.

To assess the performance of different models in water depth inversion, we also obtained the discrepancy values between the inversion results of these models and the Kriging interpolation results of the measured water depth points. The results are presented in Figure 11 and Figure 12. The discrepancy ranges of the models were compared using 2 m as the threshold. Dark blue areas indicate discrepancy values greater than 2 m, while light blue areas indicate values less than or equal to 2 m. The findings reveal that the CNN-SLI model stands out, with only 7.2% of the sea area range in large discrepancy values, which is significantly lower than the Lyzenga, MLP, and CNN models (43.3% for Lyzenga, 24.7% for MLP, and 26.8% for CNN). Therefore, through a comprehensive comparison of discrepancy maps between the inverted water depth of different models and the interpolated water depth, it is evident that the CNN-SLI model’s inverted water depth is closer to the Kriging interpolated water depth than the Lyzenga, MLP, and CNN models, indicating its superior performance.

### 4.5. Effect of Different Numbers of Training Samples on Inversion Accuracy

**Figure 13:**
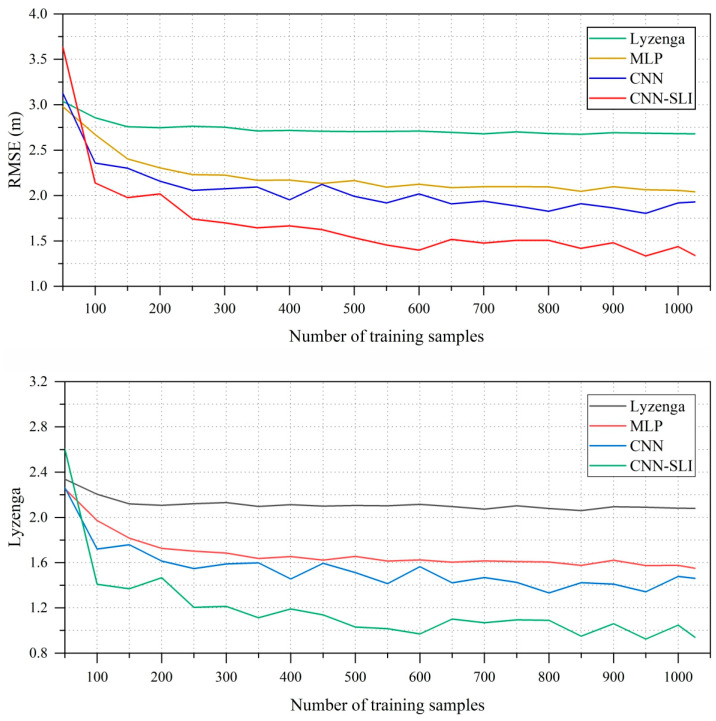
Effect of different numbers of training samples on inversion errors.

In order to achieve accurate water depth inversion, it is important to have a sufficient number of training samples. However, obtaining a significant number of training samples for real-world scenarios can be challenging. Therefore, it is crucial to carefully evaluate the impact of different numbers of training samples on the accuracy of the inversion models. To investigate this, we randomly selected different numbers of training samples ranging from 50 to 1026, with intervals of 50 (except for the final number of 1026). For each training sample number, we conducted ten independent experiments of water depth inversion and calculated the average inversion error. This average error serves as an evaluation indicator for the effectiveness of each model under the given number of training samples. The results of these experiments are presented in Figure 13.

As depicted in Figure 13, the inversion errors of the Lyzenga, MLP, CNN, and CNN-SLI models decrease significantly as the number of training samples increases until it reaches 150, 150, 100, and 250, respectively. However, the reduction effect becomes relatively limited as the number of training samples continues to increase. It is worth noting that the inversion error of the CNN and CNN-SLI models changes more with the number of training samples than that of the Lyzenga and MLP models, but when the number of training samples is not less than 100, the inversion errors of the CNN and CNN-SLI models are always lower than those of the Lyzenga and MLP models. When the number of training samples is no less than 100, the CNN-SLI model has the lowest inversion error among all models. All in all, in this study area, when the number of training samples is no less than 250, the performance advantages of the CNN-SLI model can be fully utilized, and the CNN-SLI model has the best inversion effect among all models.

### 4.6. Evaluating the Generalization Ability of Different Models

**Figure 14:**
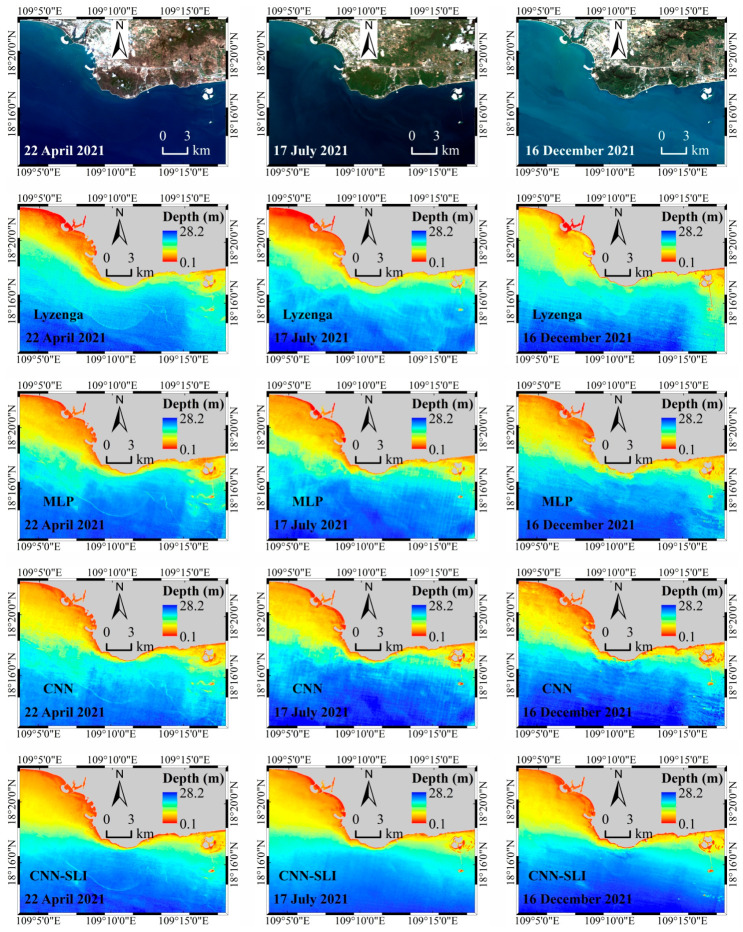
Inverted water depth maps of three GF-6 remote sensing images of Nanshan Port.

**Figure 15:**
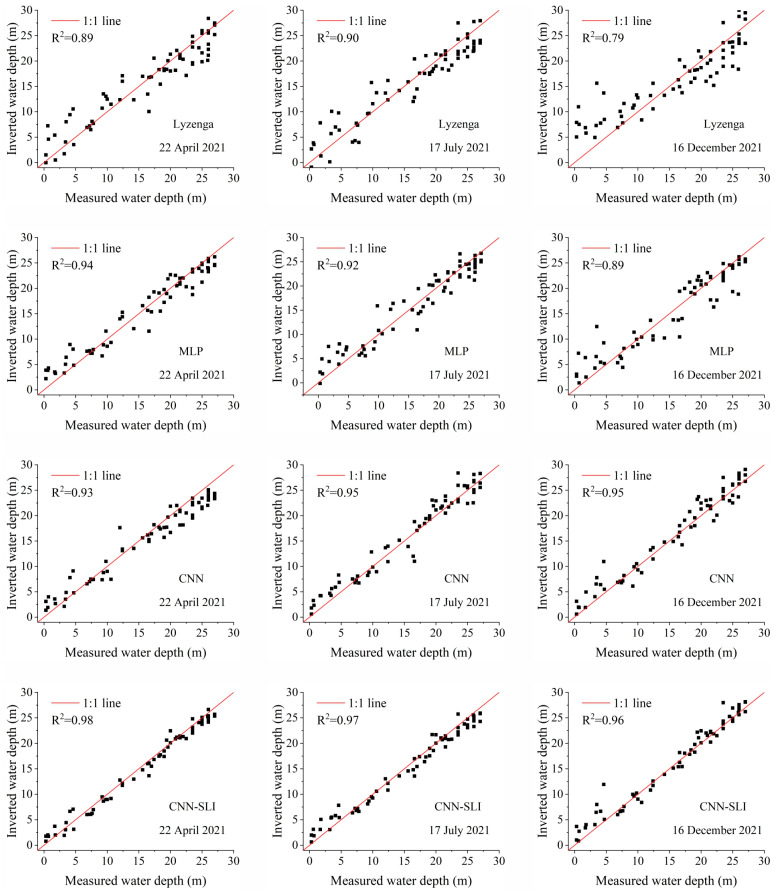
Scatter plots between the inverted water depth and measured water depth from three GF-6 remote sensing images.

In order to evaluate the generalization ability of different models, four models were trained on three preprocessed GF-6 remote sensing images (22 April 2021, 17 July 2021, and 16 December 2021) using the training samples from Dataset A, and then the trained models were used to calculate the RMSE, MAE and R^2^ on Dataset B (as an independent dataset), respectively. The RMSE, MAE, and R^2^ calculated on the independent dataset were used as the evaluation indicators of the generalization ability of the model. The water depth inversion results are shown in Figure 14, and the evaluation results are shown in Figure 15 and Table 7, Table 8 and Table 9.

As can be seen from Figure 14, the inverted water depth of the four models on the three GF-6 remote sensing images have roughly similar spatial distributions. All the inverted water depth maps show a gradual increase in water depth from the coastline to the open sea. As can be seen from Figure 15, for the three remote sensing images, the CNN-SLI model has the best regression fit, and its R^2^ is 0.96–0.98. The regression fit of the CNN-SLI model is better than that of the Lyzenga, MLP, and CNN models (the R^2^ of the Lyzenga model is 0.79–0.90, the R^2^ of the MLP model is 0.89–0.94, and the R^2^ of the CNN model is 0.93–0.95). It is obvious from the scatter distribution that the CNN-SLI model shows a higher degree of aggregation than the Lyzenga, MLP, and CNN models in almost all water depth ranges on the three remote sensing images. Table 7, Table 8 and Table 9 are the comparison results of the inversion accuracy of different models on three remote sensing images. As can be seen from Table 7, Table 8 and Table 9, for the three remote sensing images, the CNN-SLI model has significantly improved overall inversion accuracy than the Lyzenga, MLP, and CNN models. On these three remote sensing images, the CNN-SLI model has the highest overall inversion accuracy, with an RMSE of 1.18–1.70 m and an MAE of 0.99–1.19 m. It can also be seen from Table 7, Table 8 and Table 9 that for the three remote sensing images, the CNN-SLI model achieved the lowest RMSE and MAE at almost all water depth intervals. The only exception is that on the remote sensing image in December, the CNN-SLI model has higher RMSE and MAE than the CNN model at the water depth interval of 0–7 m. All in all, the CNN-SLI model has better generalization ability than the Lyzenga, MLP, and CNN models under different conditions and is more suitable for water depth inversion work in deep and shallow waters under different conditions.

### 4.7. Limitation of the CNN-SLI Model

The CNN-SLI model relies on integrating the spatial location information of pixels to deeply mine the intrinsic features of remote sensing data in the spatial dimension, and it can accurately obtain the water depth information of the waters where the training samples are located. However, the spatial portability of the CNN-SLI model may be limited due to the integration of spatial location information. For example, a model trained in “Waters A” may be difficult to migrate to “Waters B” (the substrate type and IOPs are similar to “Waters A”, but no measured water depth data are available) because it is difficult to ensure that “Waters B” has similar topographic conditions as “Waters A”. For waters not covered by training samples, the water depth inversion performance of the CNN-SLI model needs to be tested in the future.

## 5. Conclusions

This paper aims to address the issue of insufficient extraction of feature information from remote sensing data in nearshore water depth inversion research. To overcome this, the paper proposes a novel method called the CNN-SLI model, which integrates spatial location information into the convolutional neural network. By integrating the normalized spatial location information of pixels as additional channels, the model effectively captures deep features of remote sensing data in the spatial dimension, enabling accurate estimation of nearshore water depth. An experiment was conducted using GF-6 remote sensing images and measured water depth points from ENCs in the waters near Nanshan Port. The inversion results obtained using the CNN-SLI model were compared with those of the Lyzenga, MLP, and CNN models. The main conclusions are as follows:

The overall accuracy evaluation demonstrates that the CNN-SLI model outperforms the other models, with a significantly improved overall inversion accuracy and regression fit. The CNN-SLI model achieves an RMSE of 1.34 m, MAE of 0.94 m, and coefficient of determination R^2^ of 0.97. The CNN-SLI model achieves the lowest RMSE and MAE at all water depth intervals, outperforming the traditional models (Lyzenga and MLP) and the CNN model. This confirms the superiority of the CNN-SLI model for water depth inversion in shallow and deep waters. Comparative analysis with Kriging demonstrates that the inverted water depth of the CNN-SLI model is closer to the actual water depth compared to the Lyzenga, MLP, and CNN models. Moreover, in this study area, when the number of training samples is no less than 250, the CNN-SLI model fully exploits its performance advantages and exhibits the best inversion accuracy among all the models. Accuracy evaluation on an independent dataset shows that the CNN-SLI model has better generalization ability than Lyzenga, MLP, and CNN models under different conditions and is more suitable for water depth inversion work in deep and shallow waters under different conditions.

This study highlights the effectiveness of considering the spatial location information of pixels in convolutional neural networks for improving water depth inversion performance.

## Figures and Tables

**Table 1:** Key parameters of the GF-6 data used in the experiment.

Parameters	Value
Spectral range	Blue: 0.45~0.52 μm
	Green: 0.52~0.59 μm
	Red: 0.63~0.69 μm
	NIR: 0.77~0.89 μm
	Red-edge 1: 0.69~0.73 μm
	Red-edge 2: 0.73~0.77 μm
	Violet: 0.40~0.45 μm
	Yellow: 0.59~0.63 μm
Spatial resolution	16 m
Swath	800 km
Sensor	WFV
Acquisition time	17 July 2021, at 03:33:55 (UTC)
	22 April 2021, at 03:36:39 (UTC) (Only used in Section 4.6)
	16 December 2021, at 03:40:58 (UTC) (Only used in Section 4.6)

**Table 2:** Statistical information of water depth data.

Dataset	Application	Count	Maximum (m)	Minimum (m)	Mean (m)	Standard Deviation (m)
Dataset A	Training set	1026	28.2	0.1	16.87	7.79
	Validation set	440	27.6	0.2	16.76	7.66
Dataset B	Independent dataset	67	27	0.3	16.04	8.56

**Table 3:** Hyperparameter settings for the MLP model.

Hyperparameter Names	Search Space	The Values of Hyperparameters
Batch size	Integer (1, 100)	38
Learning rate	Real (1 × 10^−5^, 1 × 10^−2^)	5.76 × 10^−4^
Number of iterations	Integer (1, 2000)	524
Number of neurons in the hidden layers	Integer (4, 256)	205, 22

**Table 4:** Hyperparameters settings for the CNN model.

Hyperparameter Names	Search Space	The Values of Hyperparameters
Batch size	Integer (1, 100)	63
Learning rate	Real (1 × 10^−5^, 1 × 10^−2^)	9.35 × 10^−4^
Number of iterations	Integer (1, 200)	146
Number of convolution kernels	Integer (4, 256)	102, 10, 66, 30
Number of neurons in the fully connected layers	Integer (4, 256)	78, 153, 1

**Table 5:** Overall accuracy evaluation of water depth inversion.

Models	RMSE (m)	MAE (m)	R^2^	Running Time (s)
Lyzenga	2.68	2.08	0.88	2.44
MLP	2.04	1.55	0.93	66.89
CNN	1.93	1.46	0.94	112.12
CNN-SLI	1.34	0.94	0.97	237.84

**Table 6:** Accuracy evaluation of different models at different water depth intervals.

Models	RMSE (m)				MAE (m)			
	0–7 m	7–14 m	14–21 m	21–29 m	0–7 m	7–14 m	14–21 m	21–29 m
Lyzenga	4.22	2.49	2.24	2.28	3.20	1.92	1.88	1.89
MLP	2.46	2.38	2.28	1.40	1.68	1.77	1.92	1.12
CNN	1.71	2.12	2.39	1.51	1.24	1.47	1.94	1.22
CNN-SLI	1.57	1.66	1.21	1.12	1.05	1.06	0.87	0.88

**Table 7:** Comparison of model accuracy (22 April 2021).

Models	RMSE (m)					MAE (m)				
	Overall	0–7 m	7–14 m	14–21 m	21–29 m	Overall	0–7 m	7–14 m	14–21 m	21–29 m
Lyzenga	2.80	3.56	2.47	2.32	2.81	2.10	2.79	1.87	1.72	2.12
MLP	2.07	2.66	1.58	2.06	1.92	1.64	2.26	1.30	1.75	1.41
CNN	2.19	2.20	2.03	1.51	2.60	1.74	1.71	1.33	1.18	2.32
CNN-SLI	1.18	1.47	0.96	1.22	1.07	0.99	1.28	0.89	0.93	0.93

**Table 8:** Comparison of model accuracy (17 July 2021).

Models	RMSE (m)					MAE (m)				
	Overall	0–7 m	7–14 m	14–21 m	21–29 m	Overall	0–7 m	7–14 m	14–21 m	21–29 m
Lyzenga	2.77	3.59	2.71	2.16	2.68	2.28	3.14	2.02	1.71	2.35
MLP	2.36	2.92	2.56	2.29	1.96	1.92	2.46	1.94	2.00	1.57
CNN	1.90	2.04	1.36	2.32	1.73	1.50	1.84	1.09	1.74	1.35
CNN-SLI	1.45	1.87	0.81	1.36	1.49	1.18	1.59	0.65	1.06	1.31

**Table 9:** Comparison of model accuracy (16 December 2021).

Models	RMSE (m)					MAE (m)				
	Overall	0–7 m	7–14 m	14–21 m	21–29 m	Overall	0–7 m	7–14 m	14–21 m	21–29 m
Lyzenga	3.89	6.52	2.45	2.15	3.51	3.03	5.62	2.23	1.77	2.92
MLP	2.79	3.89	1.58	2.63	2.65	2.04	3	1.29	2.19	1.81
CNN	1.96	2.72	1.26	1.98	1.75	1.53	2.06	1.00	1.62	1.46
CNN-SLI	1.70	2.98	0.92	1.23	1.30	1.19	2.29	0.75	0.98	0.98

## Data Availability

The GF-6 WFV remote sensing image is from the China Centre for Resources Satellite Data and Application (https://data.cresda.cn, accessed on 22 March 2023). The measured water depth data are not publicly available due to private reasons.
